# Repeated mitral valve replacement in a patient with extensive annular calcification

**DOI:** 10.1186/1749-8090-6-149

**Published:** 2011-11-14

**Authors:** Tadashi Kitamura, Sachito Fukuda, Takahiro Sawada, Sumio Miura, Ikutaro Kigawa, Takeshi Miyairi

**Affiliations:** 1 Department of Cardiovascular Surgery, Mitsui Memorial Hospital, 1 Kanda Izumicho, Chiyoda, Tokyo 101-8643, Japan; 2 Department of Cardiovascular Surgery, Kitasato University Hospital, 1-15-1 Kitasato, Minami, Sagamihara, Kanagawa 252-0374, Japan; 3 Division of Cardiovascular Surgery, Totsuka Kyoritsu Second Hospital, 579-1 Yoshidacho, Totsuka, Yokohama, Kanagawa 244-0817, Japan

**Keywords:** Mitral valve replacement, Annular calcification, Surgical procedures

## Abstract

**Background:**

Mitral valve replacement in the presence of severe annular calcification is a technical challenge.

**Case report:**

A 47-year-old lady who had undergone mitral and aortic valve replacement for rheumatic disease 27 years before presented with dyspnea. At reoperation, extensive mitral annular calcification was hindering the disc motion of the Starr-Edwards mitral prosthesis. The old prosthesis was removed and a St Jude Medical mechanical valve was implanted after thorough annular debridement. Postoperatively the patient developed paravalvular leak and hemolytic anemia, subsequently undergoing reoperation three days later. The mitral valve was replaced with an Edwards MIRA valve, with a bulkier sewing cuff, after more aggressive annular debridement. Although initially there was no paravalvular leak, it recurred five days later. The patient also developed a small cerebral hemorrhage. As the paravalvular leak and hemolytic anemia gradually worsened, the patient underwent reoperation 14 days later. A Carpentier-Edwards bioprosthetic valve with equine pericardial patches, one to cover the debrided calcified annulus, another as a collar around the prosthesis, was used to eliminate paravalvular leak. At 7 years postoperatively the patient is doing well without any evidence of paravalvular leak or structural valve deterioration.

**Conclusion:**

Mitral valve replacement using a bioprosthesis with equine pericardial patches was useful to overcome recurrent paravalvular leak due to severe mitral annular calcification.

## Introduction

Severe annular calcification of the mitral valve is a major challenge to cardiac surgeons. Thorough debridement is mandatory to obtain satisfactory fitting of the prosthetic valve to the annulus. However, too much aggressive debridement can lead to atrioventricular groove perforation. Herein we present a case of prosthetic valve disorder of the mitral valve with extended annular calcification which developed 27 years after the initial surgery, requiring redo valve replacement three times to manage a paravalvular leak.

## Case Presentation

**Figure 1:**
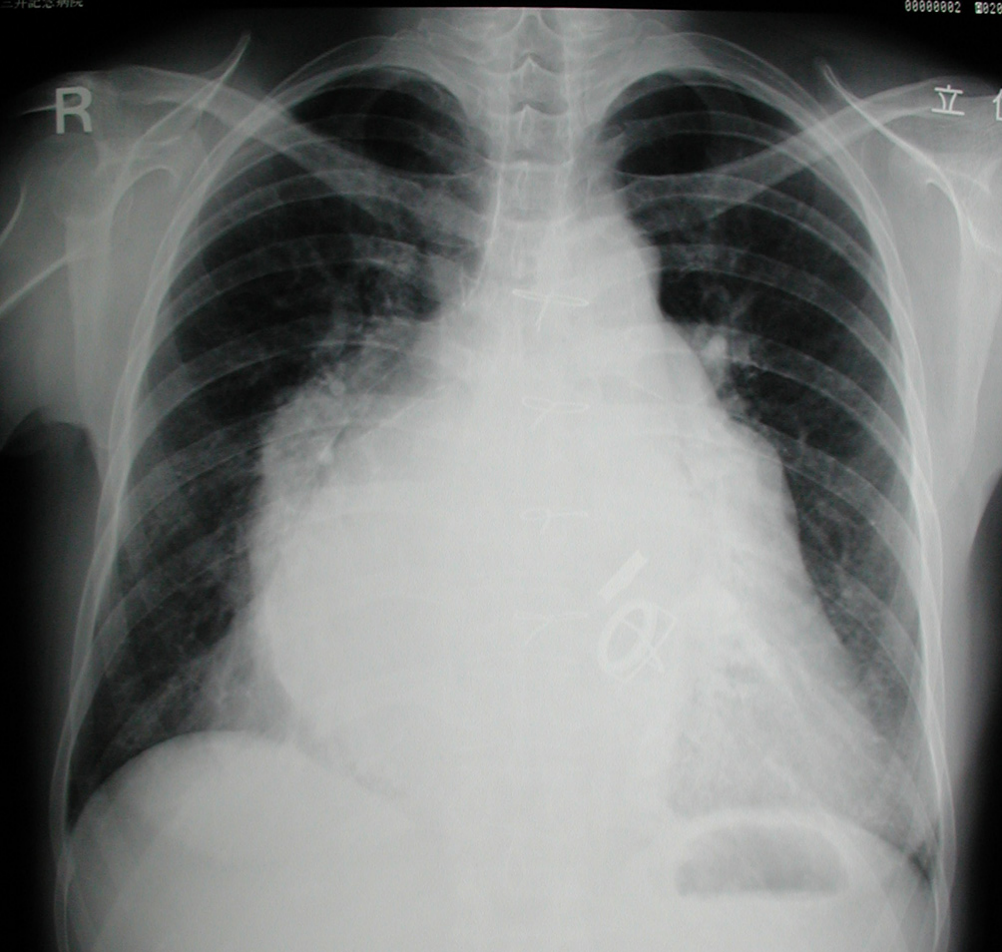
**Preoperative chest X ray showing marked dilatation of the left atrium**.

**Figure 2:**
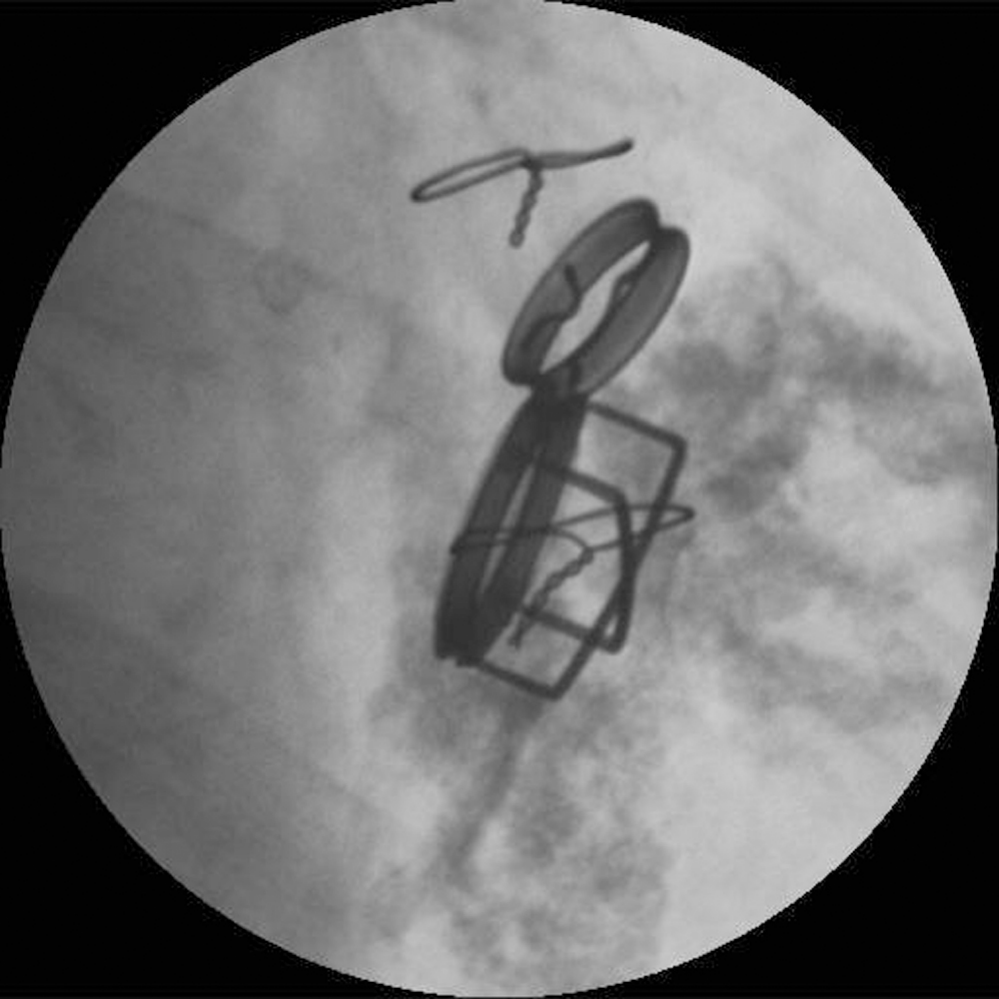
**Preoperative radiogram demonstrating severe mitral annular calcification extending to the left ventricle**.

**Figure 3:**
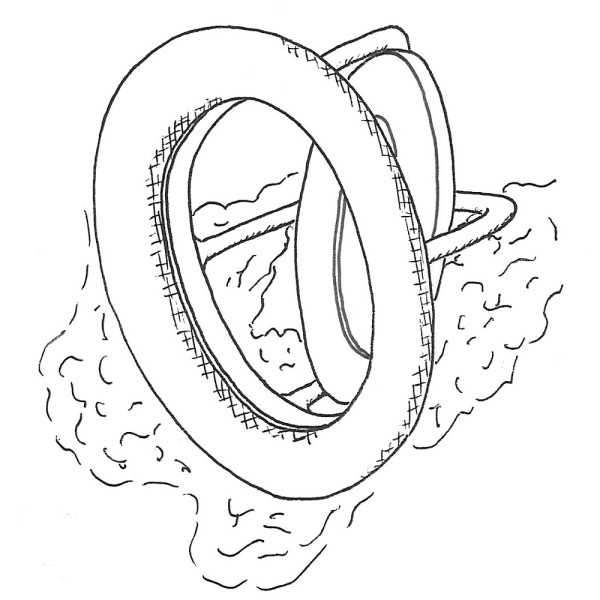
**Schematic drawing of operative findings showing extensive calcification hindering the disc motion of the prosthetic valve**.

**Figure 4:**
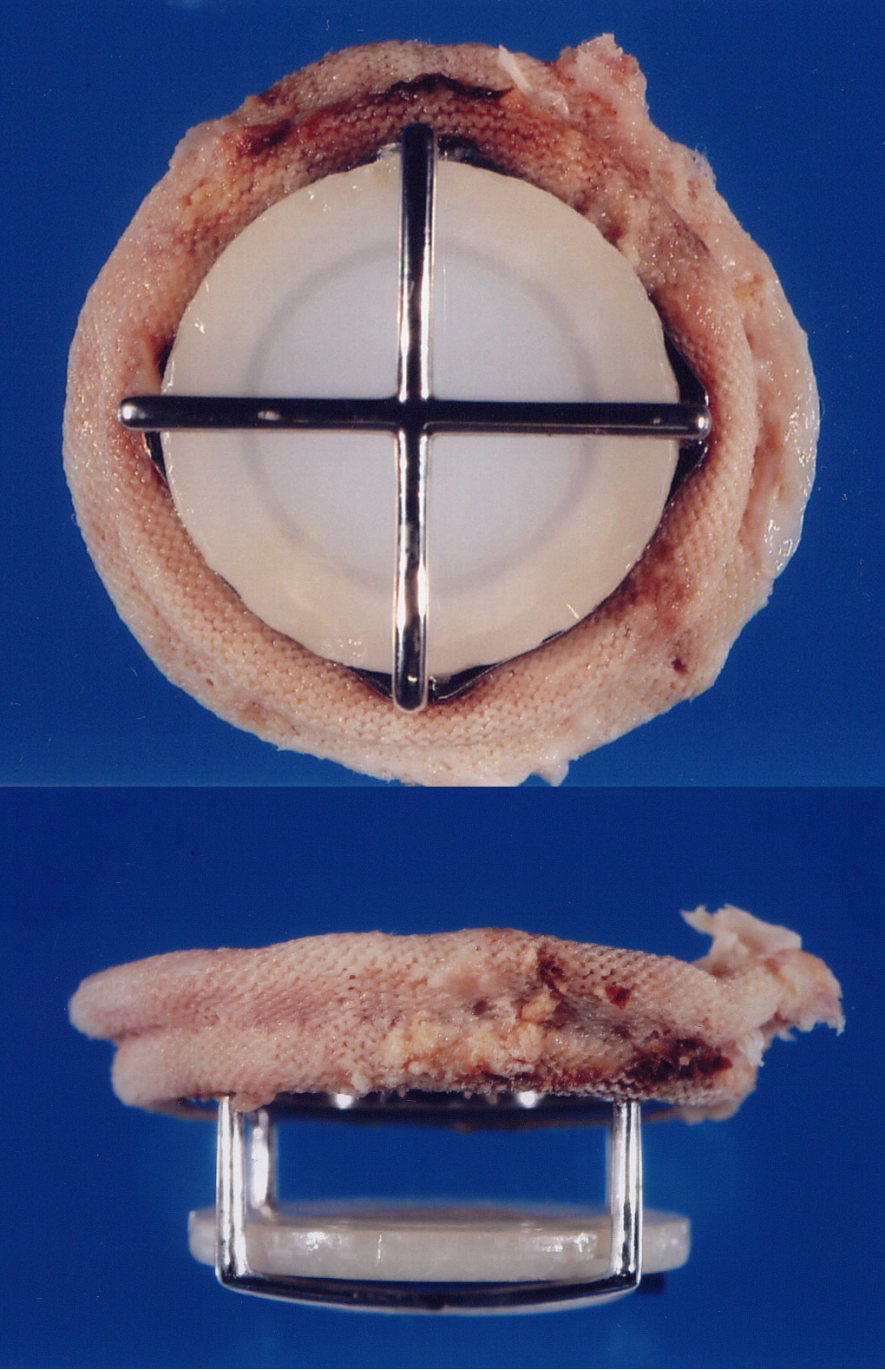
**The explanted valve showing no structural defect**.

**Figure 5:**
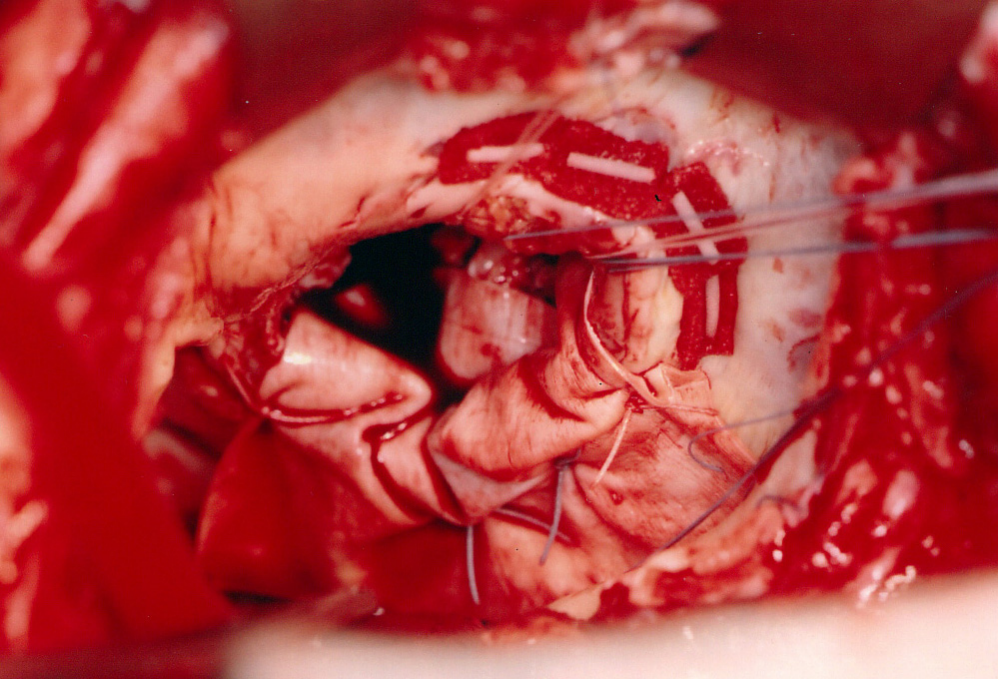
**Thoroughly debrided posterior mitral annulus covered with an equine pericardial patch**.

**Figure 6:**
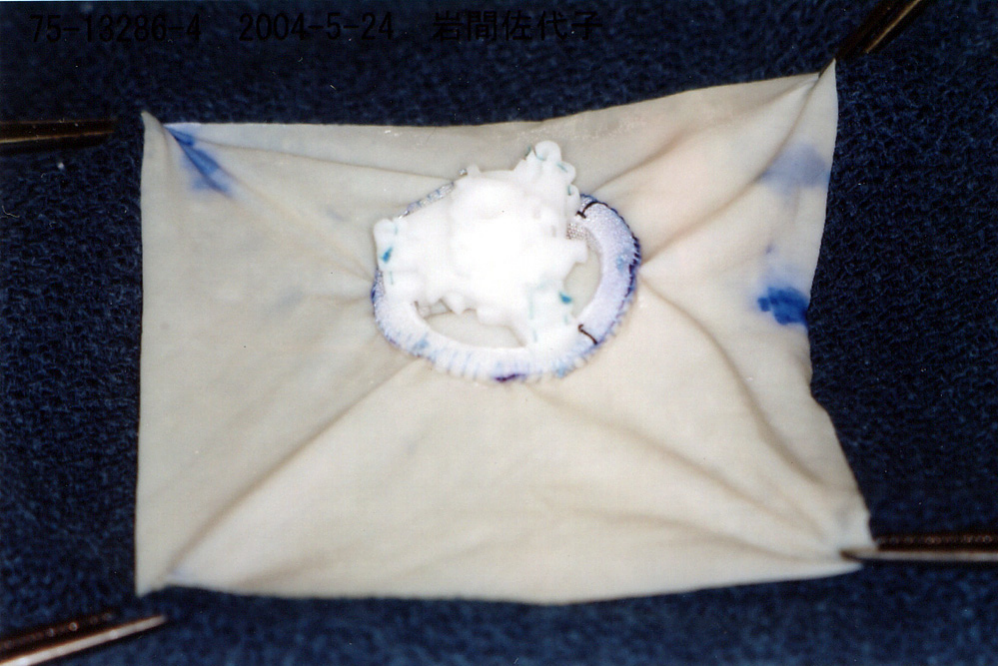
**The bioprosthetic valve sewn to an equine pericardial patch as a collar**.

**Figure 7:**
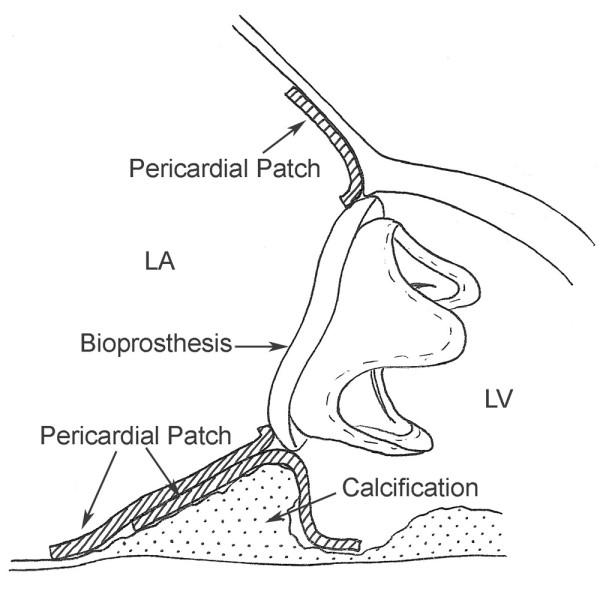
**Schematic drawing of the 2 patches, one as a caulking material, the other to obliterate the paravalvular blood flow**.

A 47-year-old lady had undergone mitral valve replacement (Starr-Edwards 6320; Edwards Lifesciences, Irvine, CA, USA) with concomitant aortic valve replacement (Bjork-Shiley; Pfizer, New York City, NY, USA) for rheumatic disease at our hospital 27 years before. On this occasion the patient developed dyspnea with New York Heart Association (NYHA) functional class II and was admitted for surgery. The chest X ray showed marked left atrial dilatation with the cardiothoracic ratio being 73% (Figure 1). The echocardiography showed that the mitral valve area measured by the pressure half time method was 1.4 cm^2 ^and that the transvalvular gradient through the aortic prosthesis was 78 mmHg. The radiography demonstrated severe mitral annular calcification extending on to the left ventricle (Figure 2). Both the mitral and aortic valve prostheses were considered to have dysfunction, therefore, replacement of the both valves was scheduled. At operation, through redo median sternotomy and right side left atriotomy, the mitral prosthesis was examined. It was evident that the calcification of the left ventricle leading to the papillary muscles was obstructing the disc motion (Figure 3). The mitral prosthesis, which had no structural defect, was removed (Figure 4) and then the aortic valve prosthesis was examined both through the aortotomy and through the left ventricle with the help of an endoscope. The aortic valve prosthesis did not have any disorder and it was left untouched. The posterior mitral annular calcification looking like a base rock was thoroughly debrided and a 27 mm mechanical valve (St Jude Medical, St Paul, MN, USA) was implanted in a paraannular position. Postoperatively transesophageal echocardiography (TEE) showed paravalvular leak and the patient developed hemolytic anemia with elevated serum lactate dehydrogenase, bilirubin and aspartate transaminase levels. Therefore, it was decided to redo the operation and a prosthesis with a heavier sewing cuff (MIRA; Edwards Lifesciences) was used this time. This reoperation was performed three days after the previous operation. A crack was found in the posterior part of the mitral annulus, and, after more aggressive debridement, a 25 mm MIRA valve was implanted in a paraannular position. Postoperatively TEE initially showed no paravalvular leak. However, a head CT demonstrated a small cerebral hemorrhage. Five days after the second operation, TEE revealed recurrent paravalvular leak which gradually worsened, and again hemolysis developed. Fourteen days after the second operation, when it was ascertained that the cerebral hemorrhage was improving, a reoperation was performed for the third time. A crack was observed at the same point in the posterior annulus and an ultrasonic aspirating device was used for further decalcification. It was concluded that the left ventricular pressure was elevated because of the pressure gradient produced by the aortic valve prosthesis. Hence a 27 mm tissue valve (Carpentier-Edwards Perimount; Edwards Lifesciences) was selected despite her age with the expectation that the risk of hemolysis would be reduced. Two equine pericardial patches were used, one on the posterior aspect of the mitral annulus for better fitting of the prosthesis (Figure 5), and the other fashioned as a collar around the prosthesis (Figure 6) to reduce paravalvular leak (Figure 7). Postoperatively TEE showed no paravalvular leak and the patient was discharged home 96 days following the procedure. At 7 years postoperatively, she has been doing well in NYHA functional class I without any evidence of paravalvular leak or structural valve deterioration.

## Discussion

Severe annular calcification is an independent risk factor for mitral valve operations [1] and can make the procedure technically challenging [2]. When the calcification process deeply involves the myocardium, sutures cannot be placed through the affected tissue. Additionally, the rough surface of the annulus can cause paravalvular leak even if a prosthesis with an expanded, heavy sewing cuff is used. On the other hand, excessive debridement may lead to atrioventricular groove perforation or injury to the circumflex artery. When there is severe annular calcification, aggressive annular debridement together with covering the annulus with a pericardial patch may be indicated to prevent paravalvular leak [3,4] because the patch simply obliterates the paravalvular blood flow and because it provides better fitting between the prosthesis and the rugged annular surface. In the present case, the left ventricular pressure was elevated due to the aortic prosthetic valve gradient. In addition, the annular calcification was so extensive and the debrided surface was so rough that paravalvular leak recurred even after the second operation. At the third operation, the risk of paravalvular leak and subsequent hemolysis was very high. Therefore, a bioprosthesis was used despite the patient's relatively young age with the expectation that the risk of hemolysis caused by the hammering effect of the mechanical valve [5] would be reduced. Placement of an equine pericardial patch eliminated the paravalvular leak. This was probably because of the "caulking" effect achieved by the patch tissue placed between the sewing cuff and the annulus, rather than by obliteration of the paravalvular blood flow by the collar sewn onto the left atrium.

## Conclusion

Mitral valve replacement with a bioprosthesis, together with the use of equine pericardial patch, could eliminate recurring paravalvular leak caused by severe mitral annular calcification extending to the left ventricle.

## Consent

Written informed consent was obtained from the patient for publication of this case report and accompanying images. A copy of the written consent is available for review by the Editor-in-chief of this journal.

## Competing interests

The authors declare that they have no competing interests.

## Authors' contributions

All authors contributed equally to the manuscript and all authors read and approved the final manuscript.
